# Genome wide association mapping and candidate gene analysis for hundred seed weight in soybean [*Glycine max* (L.) Merrill]

**DOI:** 10.1186/s12864-019-6009-2

**Published:** 2019-08-14

**Authors:** Xue Zhao, Hairan Dong, Hong Chang, Jingyun Zhao, Weili Teng, Lijuan Qiu, Wenbin Li, Yingpeng Han

**Affiliations:** 1 Key Laboratory of Soybean Biology in Chinese Ministry of Education (Northeastern Key Laboratory of Soybean Biology and Genetics & Breeding in Chinese Ministry of Agriculture), Northeast Agricultural University, Harbin, 150030 China; 2 Zhumadian Academy of Agricultural Sciences, Zhumadian, 463000 China; 3 Institute of Crop Science, National Key Facility for Crop Gene Resources and Genetic Improvement (NFCRI), Chinese Academy of Agricultural Sciences, Beijing, China

**Keywords:** Genome-wide association analysis, Hundred seed weight, Single nucleotide polymorphism, Candidate genes

## Abstract

**Background:**

The hundred seed weight (HSW) is one of the yield components of soybean [*Glycine max* (L.) Merrill] and is especially critical for various soybean food types. In this study, a representative sample consisting of 185 accessions was selected from Northeast China and analysed in three tested environments to determine the quantitative trait nucleotide (QTN) of HSW through a genome-wide association study (GWAS).

**Result:**

A total of 24,180 single nucleotide polymorphisms (SNPs) with minor allele frequencies greater than 0.2 and missing data less than 3% were utilized to estimate linkage disequilibrium (LD) levels in the tested association panel. Thirty-four association signals were identified as associated with HSW via GWAS. Among them, nineteen QTNs were novel, and another fifteen QTNs were overlapped or located near the genomic regions of known HSW QTL. A total of 237 genes, derived from 31 QTNs and located near peak SNPs from the three tested environments in 2015 and 2016, were considered candidate genes, were related to plant growth regulation, hormone metabolism, cell, RNA, protein metabolism, development, starch accumulation, secondary metabolism, signalling, and the TCA cycle, some of which have been found to participate in the regulation of HSW. A total of 106 SNPs from 16 candidate genes were significantly associated with HSW in soybean.

**Conclusions:**

The identified loci with beneficial alleles and candidate genes might be valuable for the molecular network and MAS of HSW.

**Electronic supplementary material:**

The online version of this article (10.1186/s12864-019-6009-2) contains supplementary material, which is available to authorized users.

## Background

The seed weight (SW) of soybean (often denoted by hundred seed weight (HSW)) is an important yield component and positively correlates with seed yield [1, 2]. HSW exhibits wider variation ranges [3], and the HSW of the modern elite cultivar (18–20 g) is approximately 6–7-fold greater than that of the wild soybean (3–4 g) [4]. HSW often determines the final utilization of soybean seed. Lower HSW cultivars are desirable for high quality soybean sprouts and natto production, whereas higher HSW cultivars perform well in tofu, edamame and miso production [5]. HSW also affects soybean seed germination viability and seed vigor [6]. As a typical quantitative trait, HSW is controlled by multiple genes with small or large genetic effects, especially additive effects [7, 8] and the heritability range is relatively high (44–94%). HSW is significantly affected by environmental conditions including light, temperature, soil moisture and nutrient status [9–11] and geographical conditions such as altitude, latitude, longitude and associated climate [9, 12, 13]. Although HSW has been improved from 3.5 g of wild soybean to 18–20 g of cultivated soybean through traditional methods during long-term breeding [14], breeding soybean cultivars with reasonable and stable HSW through traditional selection methods remains difficult. The traditional selection method requires evaluation in multiple environments over several years and is expensive, time-consuming and labour-intensive.

Marker assisted selection (MAS) can increase the efficiency of the traditional selection method for HSW by improving the allele frequencies of desirable HSW quantitative trait loci (QTL) [15]. Presently, linkage analysis, based on special bi-parental mapping populations, is still extensively applied to dissect the genetic base (or QTL) of HSW. More than 200 QTL have been reported in the SoyBase databank (www.soybase.org), which are distributed on 20 chromosomes (Chr, or linkage group (LG)) from more than 40 different genetic populations and 50 parental materials. Most of these identified QTL were found in F_2_ or recombinant inbred line (RIL) populations [7, 16–26]. Except for these identified QTL in mature seeds, some studies analysed the dynamic QTL of HSW during the different developmental stages, which have increased the understanding of HSW QTL [19, 27]. Only a few genes controlling HSW of soybean, have been cloned till date. A phosphatase 2C-1 (PP2C-1) gene was found to contribute to the increase of HSW in transgenic plants through a combination of whole-genome sequencing and an RIL population derived from a cross between a wild soybean ZYD7 and a cultivated soybean HN44 [28]. Among these identified QTL, most spanned fairly large genomic regions due to the relatively low density of molecular markers (http://www.soybase.org), which have a relatively low accuracy, limiting their application in MAS efforts.

Genome-wide association studies (GWAS) have more extensive recombination events and shorter linkage disequilibrium (LD) blocks due to high density of SNP markers used for mapping and wider phenotypic variation available in germplasm. Therefore, GWAS could significantly improve the resolution and accuracy of marker-phenotype associations compared with linkage analysis, based on bi-parental mapping populations. Presently, GWAS have begun to effectively analyze the genetic basis of HSW in soybean. Yan et al. identified 17 HSW QTL on six chromosomes through 166 samples with SoySNP50K BeadChip based on two tested environments. Zhang et al. found a total of 22 HSW QTL with minor effects based on 309 germplasm accessions and 31,045 polymorphic SNPs [29, 30]. However, as of late 2018, no studies have identified QTL underlying HSW of soybean in Northeast China based on sequencing technology.

In the present study, we performed a GWAS of soybean HSW in Northeast China based on 185 tested accessions and 24,180 SNPs. The aim of the present study is to identify QTLs associated with HSW and to screen candidate genes located in peak SNP regions.

## Result

### Distribution of hundred seed weight in the association panel

**Fig. 1:**
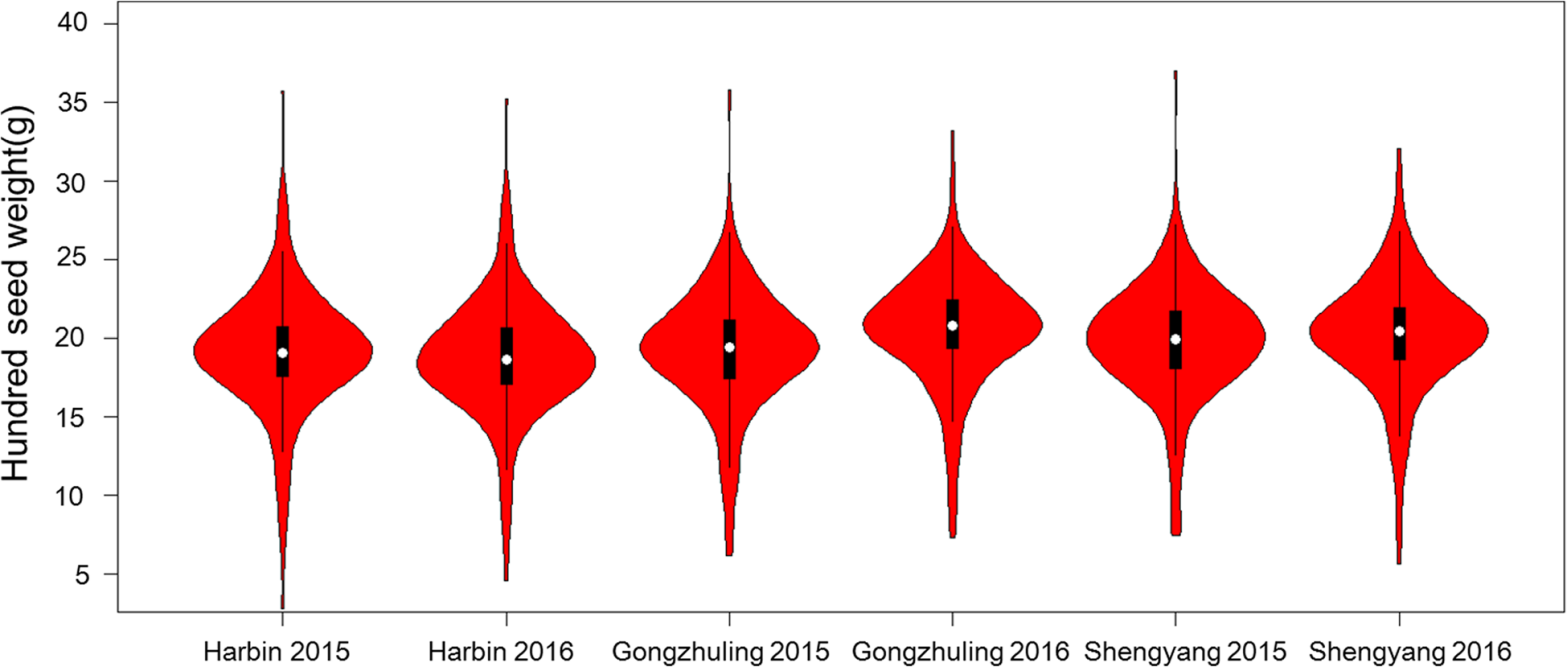
Variation of hundred seed weight of soybean in the association panel

The phenotypic value was evaluated in three locations for two years to analyse the variation distribution (Additional file 1). The association panel consisted of 185 accessions with HSW varying from 5.64 g to 34.8 g, and an average HSW of 19.60 g was calculated based on the mean observed across the three tested environments in 2015 and 2016 (Additional file 1). Coefficients of variation ranged from 18 to 21% for the different environments, and significant differences among the tested environments were not found for the association panel (Additional file 1). Normal distributions without any significant skewness and kurtosis were observed for the association panel in all tested environments (Fig. 1 and Additional file 1).

### Distribution of SNPs and genetic characteristics of the mapping population

**Fig. 2:**
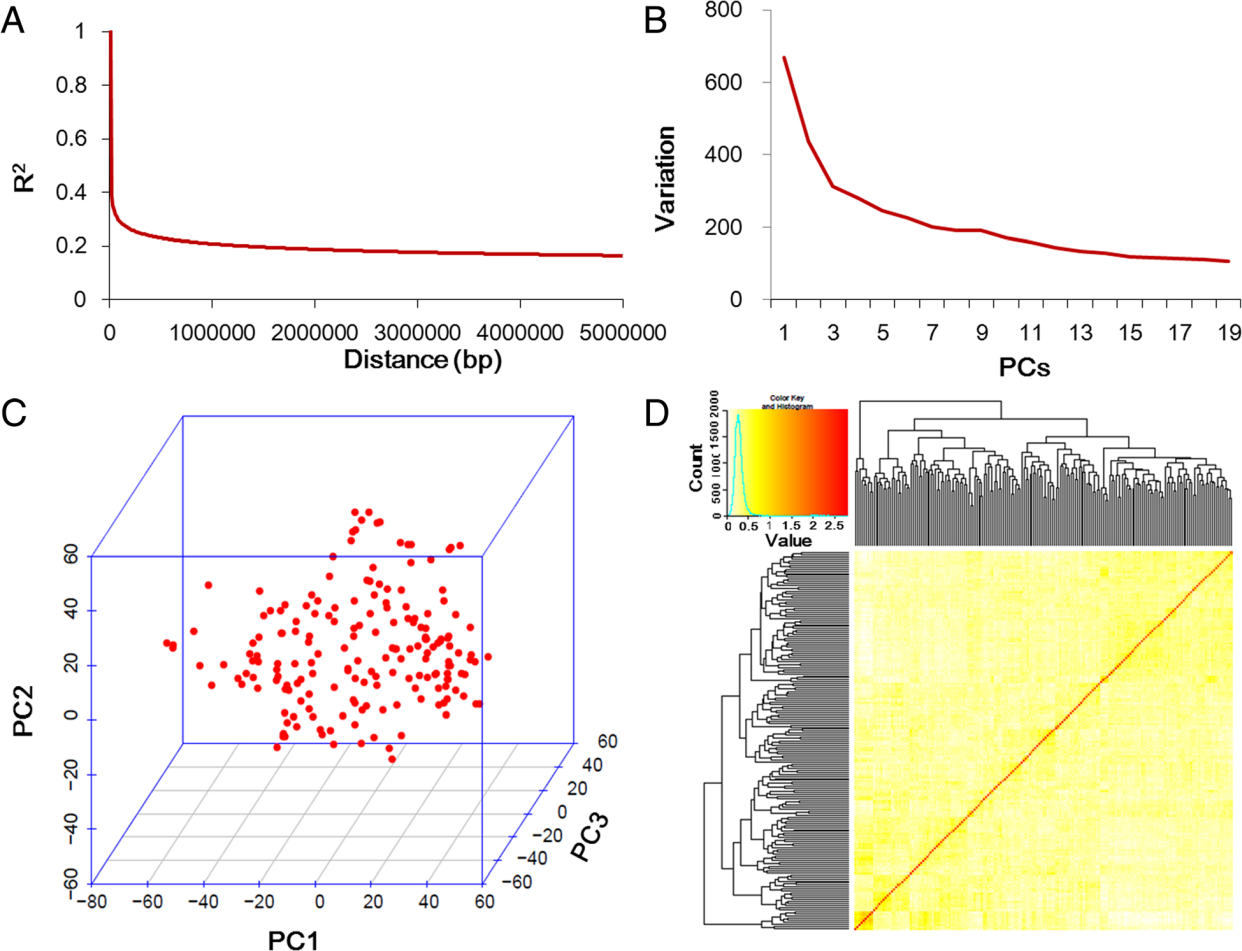
Linkage disequilibrium (LD) evaluation and genetic features of the mapping population. **a** LD decay of the genome-wide association study (GWAS) population. **b** The first three principal components reflected by SNPs used in the GWAS. **c** Population structure of soybean germplasm. **d** A heatmap of the kinship matrix of the 185 soybean accessions

A total of 24,180 SNPs distributed across all 20 chromosomes of the soybean genome with minor allele frequencies > 0.05 and missing data less than 3% were utilized to estimate LD levels in the 185 tested samples. These SNP markers spanned 947.89 Mbp, which accounted for approximately 86.17% of the entire soybean genome. The number of SNPs among the 20 chromosomes was not even, and large variations in the number of SNPs (from 676 in Chr.11 to 1774 in Chr.15) were observed among different chromosomes. The average marker density was approximately 1 SNP per 40.29 kb (Table 1). The decay distance of the LD between markers was 215.74 kb (Fig. 2a).

**Table 1:** Number and density of single nucleotide polymorphisms (SNPs) on each chromosome for the genome-wide association study (GWAS)

Chromosome number	Number of SNPs^a^	Sequence length (Mb)	SNP density (Kb/SNP)
1	1278	56.82	44.46
2	1128	48.47	42.97
3	1146	45.73	39.9
4	1431	52.32	36.56
5	862	42.17	48.92
6	1336	51.3	38.4
7	1082	44.6	41.22
8	1092	47.8	43.77
9	1312	50.18	38.25
10	1241	51.55	41.54
11	676	34.7	51.33
12	808	40.07	49.59
13	1261	45.6	36.16
14	1078	48.99	45.45
15	1774	51.67	29.13
16	1168	37.8	32.36
17	1185	41.61	35.11
18	1739	58.01	33.36
19	1463	50.6	34.59
20	1120	47.9	42.77

^a^ single nucleotide polymorphism

### Quantitative trait nucleotide (QTN) associated with hundred seed weight by GWAS

**Fig. 3:**
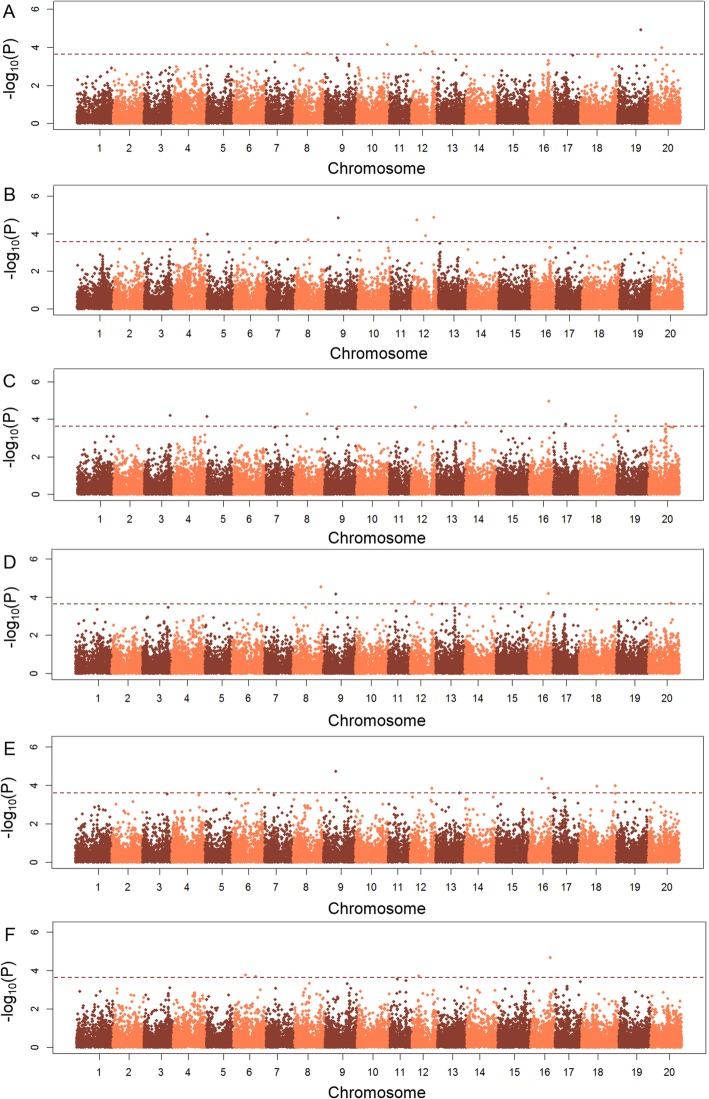
Manhattan plot of association mapping of hundred seed weight in soybean. **a**-**b**: Harbin in 2015 and 2016; **c**-**d**: Gongzhuling in 2015 and 2016; **e**-**f**: Shenyang in 2015 and 2016. The dashed line on each subgraph indicated the log10 (*p* Value) significance threshold

A compressed mixed linear model (CMLM) was used to identify association signals using the R package GAPIT. Principal component and kinship analyses of the association panel were evaluated using the whole set of SNPs, which were also considered in the CMLM model. The first three principle components (PCs) accounted for 12.48% of the total genetic variation (Fig. 2b-c). The evaluation of the variation of the first 20 PCs analysis revealed an inflection point at PC3 (Fig. 2b), suggesting that the first three PCs dominated the population structure on the association mapping. A lower genetic relatedness within the population was exhibited from the distribution of the pairwise relative kinship coefficients of the association panel among the 185 tested accessions (Fig. 2d). In the present study, thirty-four association signals were associated with HSW, which were distributed on fifteen chromosomes, including Chr.3, Chr.4, Chr.5, Chr.6, Chr.8, Chr.9, Chr.10, Chr.12, Chr.13, Chr.14, Chr.16, Chr.17, Chr.18, Chr.19, and Chr.20 (Fig. 3 and Table 2). Among them, five SNP loci (HSW-8-1 on Chr.8, HSW-9-1 on Chr.9, HSW-12-1 on Chr.12, HSW-12-4 on Chr.12, and HSW-16–3 on Chr.16) were identified in more than three environments in this study. Another thirty QTNs were found in less than three environments. Among all 34 QTNs, fifteen signals overlapped or located near the genomic region of the known QTL underlying HSW or soybean yield, and the other seventeen were novel for HSW (Table 2). The HSW of the tested accessions with different alleles were evaluated, and the results indicated that the HSW of these accessions with different alleles in these identified QTNs were significantly different (Table 2). Thus, utilization of these appropriate alleles for HSW would be interesting for MAS of soybean cultivars with higher HSW.

**Table 2:** Single nucleotide polymorphisms (SNPs) associated with hundred seed weight of soybean and known QTL overlapped with peak SNP

Locus name	Environment^al^	Chr.^b^	Position	Alleles	Allelic effect	-Log_10_(P)	MAF	R^2^(%)	Known QTLs
HSW-3-1	E3	3	40,302,935	G:T	2.25	4.2	0.07	31.96	Seed weight per plant 5-3_40168335–42,675,829 (Kuroda et al. 2013); Seed yield 27-4_40375902–41,065,116 (Kim et al. 2012); Seed yield 30-4_40375902–41,065,116 (Kim et al. 2012);
HSW-4-1	E2	4	33,447,909	A:G	2.3	3.7	0.14	31.85	Seed width 1-10_32617784–45,860,827 (Salas et al. 2006)
HSW-4-2	E2	4	33,617,714	A:G	2.3	3.7	0.14	31.93	Seed width 1-10_32617784–45,860,827(Salas et al. 2006)
HSW-5-1	E2, E3	5	431,686	A:G	−1.68/− 1.61	4.15/3.97	0.14	32.33/32.11	
HSW-5-2	E5	5	38,064,280	G:T	1.01	3.58	0.28	32.49	
HSW-6-1	E6	6	18,590,024	G:T	1.59	3.77	0.09	34.19	
HSW-6-2	E6	6	34,877,639	A:G	1.99	3.68	0.07	33.33	
HSW-6-3	E5	6	41,987,021	G:T	1.77	3.79	0.08	32.85	
HSW-8-1	E1, E2, E3	8	20,122,716	G:T	1.84/1.7/1.64	4.28/3.69/3.67	0.10	33.22/32.46/32.64	
HSW-8-2	E2	8	43,658,396	A:G	−2.36	4.53	0.07	32.45	
HSW-9-1	E2, E4, E5	9	19,237,332	G:T	1.8/2.05/1.98	4.73/4.14/4.84	0.09	30.85/31.95/31.18	
HSW-10-1	E1	10	48,019,613	A:G	−1.64	4.12	0.13	33.4	Seed weight per plant 3-2_47716772–48,485,990 (Liu et al. 2011)
HSW-12-1	E1, E2, E3, E4	12	6,618,366	C:T	2.32/1.93/2.17/2.29	4.64/4.07/3.76/4.71	0.07	33.5/32.43/31.92/31.07	Seed weight 23–2_6653096–7,980,959 (Li et al. 2008)
HSW-12-2	E6	12	10,343,129	G:T	1.56	3.71	0.12	32.28	
HSW-12-3	E1, E2	12	19,897,222	A:G	−1.92/−1.93	3.67/3.88	0.10	32.66/31.39	
HSW-12-4	E1, E2, E4	12	32,409,801	A:C	−1.94/−2.15/−2.43	3.77/3.54/4.85	0.06	31.91/32.68/34.9	
HSW-12-5	E5	12	33,768,654	A:T	1.31	3.85	0.20	33.03	
HSW-13–1	E4	13	10,148,283	C:T	2.4	3.64	0.07	31.05	
HSW-13 − 2	E3	13	29,533,558	A:C	1.31	3.64	0.22	30.71	Seed yield 28–11_29609521–32,196,800 (Rossi et al. 2013); Seed weight 40-1_29609521–32,196,800 (Rossi et al. 2013); Seed weight 49–13_29609521–31,661,129 (Teng et al. 2009)
HSW-13-3	E5	13	37,094,696	G:T	1.81	3.59	0.07	30.71	Seed weight 45-6_32,196,800–39,208,429 (Yan et al. 2014)
HSW-14–1	E3, E4	14	981,334	A:G	-2/−1.81	3.81/3.54	0.07	34.09	Seed weight 29–1_439027–971,657 (Liu et al. 2011)
HSW-16–1	E5	16	20,127,714	A:G	−2.11	4.34	0.07	34.6	Seed weight 30-6_16724085–27,167,274 (Kim et al. 2010)
HSW-16–3	E3, E4, E5, E6	16	30,250,524	G:T	1.94/1.66/1.6/1.76	4.96/3.84/4.18/4.68	0.10	35.54/35.39/34.04/33.33	
HSW-17-1	E5	17	1,004,800	A:G	3	3.57	0.10	32.55	Seed weight 3–1_961346–2,201,427 (Mian et al. 1996)
HSW-17-2	E3	17	19,283,709	C:T	1.66	3.75	0.10	36.28	
HSW-17-3	E1	17	29,346,634	A:G	−2.15	3.58	0.06	31.8	Seed weight 34–17_24110077–37,831,244(Han et al. 2012)
HSW-18–1	E5	18	27,066,313	G:T	−2.11	3.96	0.07	36.03	Seed weight per plant 6-7_22375695–48,185,138 (Yao et al. 2015)
HSW-18–2	E3	18	56,307,027	C:G	1.65	3.92	0.12	35.43	
HSW-18-3	E3,E5	18	56,316,047	G:T	−1.65	4.18/3.97	0.14	34.42/31.38	
HSW-19–1	E1	19	36,849,383	A:C	−2.27	4.91	0.07	36.31	
HSW-20-1	E1	20	19,267,460	C:T	2.24	3.97	0.07	31.57	Seed yield 9–1_3903416–27,664,504 (Yao et al. 2015)
HSW-20-2	E3	20	26,794,777	C:T	−1.38	3.74	0.31	31.37	Seed yield 10–1_2716974–25,498,552 (Yao et al. 2015)
HSW-20-3	E3,E4	20	35,358,859	A:G	−1.11/− 1.07	3.57/3.67	0.46	32.63/37.25	Seed weight 36-5_34302228–46,787,225(Han et al. 2012)
HSW-20-4	E3	20	37,897,358	G:T	−1.95	3.57	0.07	31.62	

^a^E1: at Harbin in 2015, E2:at Harbin in 2016, E3: at Gongzhuling in 2015, E4: at Gongzhuling in 2016, E5: at Shenyang in 2015, E6 at Shenyang in 2016; ^b^ Chromosome

### Prediction of candidate genes controlling hundred seed weight

Genes located in the 200-kbp flanking regions of each peak SNP were considered candidate genes. A total of 237 genes, derived from 31 QTNs, were located near peak the SNPs from the three tested environments in 2015 and 2016 (Additional file 2). For further clearing the potential functions of these genes, various functional groups were classified based on the Gene Ontology database (http://geneontology.org/). Of these inferred genes, fifty-four had no functional annotations and were derived from protein families with unknown function. Another 183 genes were related to plant growth regulation, hormone metabolism, cell, RNA, protein metabolism, development, starch accumulation, secondary metabolism, signalling, and the TCA cycle (Additional file 2). Among these identified candidate genes, *Glyma.03G192300*, a starch branching enzyme located near HSW-3-1 of Chr.3, could promote seed weight in crops [31]. *Glyma.05G005400* (located near HSW5–1 of Chr.5) and *Glyma.08G317300* (located near HSW8–2 of Chr.5), a MYB-domain protein, play key roles in regulating seed development and determining seed weight in plants [32]. *Glyma.05G196400* (located near HSW5–2 of Chr.5), one Leucine-rich repeat protein kinase family protein, has been proven a regulator of seed weight in plants [33]. Masatake et al. (2013) reported that HS3, which has the same domain as *Glyma.06G201700* (located near HSW6–1 of Chr.6), regulates seed development in plants [34]. Bhatnagar et al. (2017) reported that *OsPP2C51* positively regulates rice seed germination and affects seed yield [35]. Both *OsPP2C51* and *Glyma.12G109500* (located near HSW-12-2 of Chr.12) are members of protein phosphatase 2C.

**Fig. 4:**
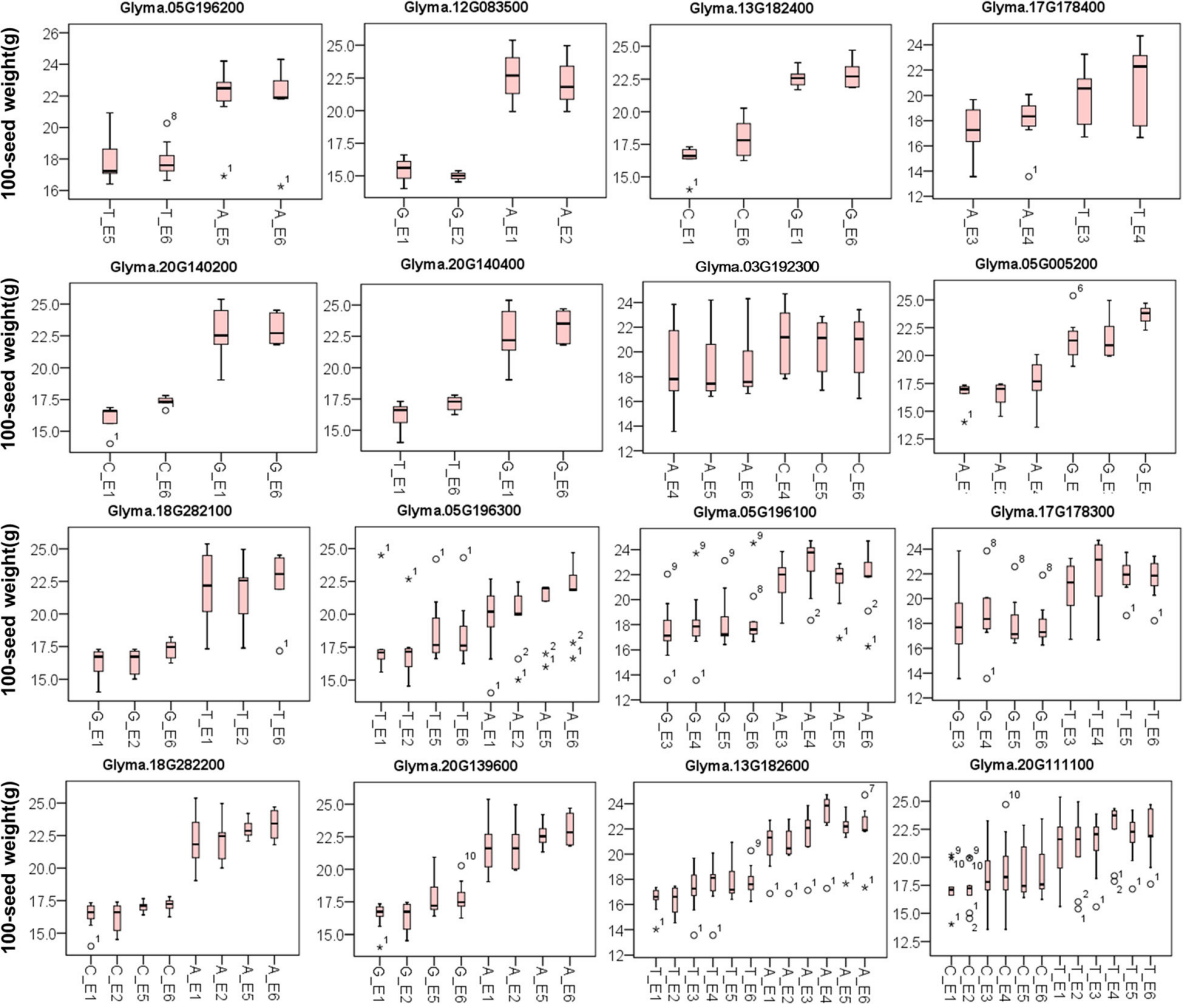
Haplotypes analysis of genes with variations related to HSW. E1 and E2: Harbin in 2015 and 2016; E3 and E4: Gongzhuling in 2015 and 2016; E5 and E6: Shenyang in 2015 and 2016. In each subgraph, the symbols ‘^*^’ and ‘^**^’ next to gene IDs represent the suggested significance of the t-test at *p* < 0.05 and *p* < 0.01, respectively, “^o^” represents mild outliers, “*” represents extreme outliers and error bars represent Std. Deviation

To identify the possible roles of candidate genes in the HSW of soybean, gene-based associations were conducted using the GLM method. A total of 3057 SNPs in 237 candidate genes (MAF > 0.10) were obtained among twenty lines (ten higher/lower HSW lines) through genome re-sequencing. A total of 106 SNPs from 16 genes (*Glyma.05G196200, 4 SNP*; *Glyma.12G083500, 4 SNP*; *Glyma.13G182400, 4 SNP*; *Glyma.17G178400, 4 SNP*; *Glyma.20G140200, 4 SNP*; *Glyma.20G140400, 4 SNP*; *Glyma.03G192300, 6 SNP*; *Glyma.05G005200, 6 SNP*; *Glyma.18G282100, 6 SNP*; *Glyma.05G196300, 8 SNP*; *Glyma.05G196100, 8 SNP*; *Glyma.17G178300, 8 SNP*; *Glyma.18G282200, 8 SNP*; *Glyma.20G139600, 8 SNP*; *Glyma.13G182600, 12 SNP*; *Glyma.20G111100, 12 SNP*) were significantly associated with HSW in soybean (Fig. 4, Additional file 3). Among these 16 identified genes, one gene (*Glyma.03G192300*) has been reported to be associated with HSW in crops [31]. The other 15 genes were found to be novel for controlling SW in soybean. The two alleles of the peak SNP from each of the 16 genes were analyzed for allelic effects. The SW of soybean accessions with one allele was significantly different from the other for all the 16 SNP peaks (Fig. 4). These beneficial alleles from candidate genes would be helpful for MAS in soybean with high and stable HSW.

## Discussion

As the important source for vegetable oil and plant protein, soybean yield is lower in comparison with other major crops. MAS usefulness and effectiveness of a crop depends on two key factors: the accuracy of QTNs and the genetic variation in the selected association panel. The more diverse of the selected association panel in genotype and phenotype, the better universal of QTNs and beneficial alleles obtained by genome-wide association analysis as molecular markers for molecular assisted selection breeding. Soybean was difficult to adapt and fully mature in the unadoptable environments because it has a significant photoperiod response [30]. In this study, a total of 185 landraces or elite cultivars that were derived from six maturity groups (maturity groups 000, 00, 0, I, II and III) [14] were used to evaluate phenotypic variation (Additional file 4). The samples from these six maturity groups were selected since the accessions from different maturity groups have special adaptability to the three tested sites of Northeast China (‘Harbin’, ‘Gongzhuling’ and ‘Shenyang’) with two degrees difference in latitude between each two sites, which could fully reflect the formation of HSW and accurately evaluate the effects of environment on HSW to effectively increase the accuracy of the phenotypic data and QTNs. A previous study showed that environmental factors significantly affected the stability of HSW, especially in different maturity group conditions [9, 12, 13]. In the preset study, the HSW of most tested samples behaved stable among the three tested environments, which indicated that the variation of HSW was relatively smaller in the appropriate environment (Additional file 1). Zhang et al. (2015) proposed a new way to incorporate a superior allele of each QTN to improve the efficiency and accuracy of traditional HSW breeding, which was valuable for MAS of HSW in Northeast China [36].

To date, more than 200 HSW QTL have been identified based on different mapping populations through bi-parental hybrid population, and most of these identified QTL were specific to the genetic background. Therefore, the novel QTL/genes still need to be identified for HSW MAS. In this study, 34 QTNs located on 15 chromosomes were found that are associated with HSW in three environments in 2015 and 2016. Among these 34 association signals, fifteen QTNs overlapped with or near the known HSW QTL (Table 2). Two QTNs (HSW-3-1 of Chr.03 and HSW-13-2 of Chr.13) were significantly associated with HSW, and the association between these two genomic regions and HSW had been repeatedly verified by many previous linkage analysis studies [8, 27, 37, 38]. Similarly, twelve QTNs (HSW-4-1 and HSW-4-2 of Chr.04, HSW-10-1 of Chr.10, HSW-13-3 of Chr.13, HSW-14–1 of Chr.14, HSW-16–1 of Chr.16, HSW-17-1 and HSW-17-3 of Chr.17, HSW-18–1 of Chr.18, HSW-20-1, HSW-20-2 and HSW-20-3 of Chr.20) have also been reported [4, 16, 20, 39–41]. Li et al. (2008) identified one major QTL (Seed weight 23–2 located in Chr.12) that affects HSW using a wild soybean-derived mapping population, which has a similar genomic region to HSW-12-1 in this study [42]. Another 18 QTL that are novel for HSW were identified.

Presently, only a few genes that control HSW were identified. Lu et al. (2017) discovered that a phosphatase 2C-1 (PP2C-1) allele from wild soybean ZYD7 contributes to the increase in HSW [28]. GWAS could offer some valuable clues to identify and validate the candidate genes of HSW, especially in the relatively smaller LD block (average length 150–200 kbp) of soybean. Among these identified candidate genes, *Glyma.03G192300* (located near HSW-3-1 of Chr.3), *Glyma.05G005400* (located near HSW5–1 of Chr.5), *Glyma.08G317300* (located near HSW8–2 of Chr.5), *Glyma.05G196400* (located near HSW5–2 of Chr.5), *Glyma.06G201700* (located near HSW6–1 of Chr.6), and *Glyma.12G109500* (located near HSW-12-2 of Chr.12) have been reported to play key roles in regulating seed development and determining seed weight in crops, specific mechanisms of which still need to be analysed.

## Conclusions

By performing a GWAS of soybean HSW in Northeast China based on 185 tested accessions and 24,180 SNPs, the results showed that 34 QTNs located on 15 chromosomes are associated with HSW in three environments for two years. Among these 34 association signals, fifteen QTNs overlapped with or near the known HSW QTL. Gene based association showed 106 SNPs from sixteen candidate genes were significantly associated with HSW in soybean. Of them, *Glyma.03G192300* was previously reported to be important in seed development and the other 15 gene were novel genes for HSW in soybean. The present study provides an invaluable resource and new QTL/genes for further study on the HSW of soybean in the molecular network and molecular assistant selection.

## Methods

### Soybean germplasms and field trials

One hundred and eighty-five samples were selected and collected from the Chinese National Soybean GeneBank to analyse the HSW variation and for subsequent reduced-sequencing. Among these accessions, there were 97 elite varieties, 38 elite lines and 28 landraces from the soybean production areas between 36.23° N and 61.50° N of China belonged to six maturity groups (maturity groups 000, 00, 0, I, II and III) [14], representing the geographical and ecological diversity of soybean in northern China (Additional file 4). The other 22 accessions were collected from non-Chinese regions. All plant materials tested were planted at three locations including Harbin (45.80° N, 126.53° E, chernozem, active accumulated temperature 2700 °C, frost-free period is 135 d, annual precipitation is 500–600 mm), Gongzhuling (43.50° N, 124.82° E, chernozem, active accumulated temperature is around 3010 °C, frost-free period is 144 d, annual average precipitation is 500–700 mm), and Shenyang (41.80° N, 123.38° E, chernozem, active accumulated temperature is around 4010 °C, frost-free period is 155 d, annual precipitation is 500–800 mm) in 2015 and 2016. For the tested environments, field trials were performed with a single row plot (3 m long and 0.65 m between rows) based on randomized complete block design and three replicates. After reaching full maturity of all plant materials, a total of 10 randomly selected plants from each row in each plot were randomly picked and weighed and the HSW was evaluated.

### DNA isolation and SNP genotyping data collection

Genomic DNA of tested samples was isolated via the hexadecyl trimethyl ammonium bromide (CTAB) method and genotyped through a reduced-sequencing method (the specific locus amplified fragment sequencing (SLAF-seq) methodology) [43]. Two restriction digestion enzymes, *Mse*I (EC 3.1.21.4) and *Hae*III (EC: 3.1.21.4) (Thermo Fisher Scientific Inc., Waltham, MA, USA), were selected to produce more than 50,000 sequencing tags (approximately 300 bp to 500 bp in length) in each tested sample. In each accession, the sequencing libraries were defined based on the obtained sequencing tags, which spanned unique genomic regions in soybean. The barcode method and Illumina Genome Analyzer II System (Illumina Inc., San Diego, CA, USA) were utilized to obtain the 45-bp sequence reads at both ends of the sequencing tags from each accession library. The alignment between the obtained raw paired-end reads and the reference genome was conducted with BWA software (Version: 0.6.1-r104) [44]. The raw reads in the same genomic position were used to define the SLAF groups using more than 58,000 high-quality SLAF tags from each tested sample. The SNPs were defined based on an MAF ≥ 0.05. The genotype was regarded as heterozygous when the depth of minor allele/the total depth of the sample ≥ 1/3 (Additional file 5).

For twenty lines with extreme phenotypic values of SW, a genome resequencing with 10-fold in depth was conducted on an Illumina HiSeq 2500 sequencer. Paired-end resequencing reads were mapped to the reference genome (Version: Glyma.Wm82.a2) with BWA (Version: 0.6.1-r104) using the default parameters. SAMtools48 (Version: 0.1.18) software was used to convert the mapping results into the BAM format and to filter the unmapped and non-unique reads. Duplicated reads were filtered with the Picard package (picard.sourceforge.net, Version: 1.87). The BEDtools (Version: 2.17.0) coverage Bed program was applied to compute the coverage of sequence alignments. A sequence was defined as absent when coverage was lower than 90% and present when coverage was higher than 90%. SNP detection was performed by the Genome Analysis Toolkit (GATK, version 2.4–7-g5e89f01) and SAMtools. Only the SNPs detected by both methods could be analyzed further. SNPs with allele frequencies lower than 1% in the population were discarded (Additional file 6). SNP annotation was performed based on the reference genome (Version:Glyma. Wm82.a2) using the package ANNOVAR (Version: 2013–08–23).

### Population structure evaluation and linkage disequilibrium (LD) analysis

The principal component analysis (PCA) programs were used to analyze the population structure of the association panel through GAPIT software [45]. The squared allele frequency correlations (r^2^) in TASSEL version 3.0 [46] were used to calculate the LD block across the soybean genome based on SNPs with MAF ≥ 0.05 and missing data ≤10%. In contrast to the GWAS, missing SNP genotypes were not imputed with the major allele before LD analysis. The parameters in the software programs were set with MAF (≥ 0.05) and the integrity of each SNP (≥ 80%).

### Genome-wide association analysis

Association signals of HSW were identified based on 24,180 SNPs from 185 tested samples with the compressed mixed linear model (CMLM) with default parameters in GAPIT [45]. The *P* value was calculated with the Bonferroni method with α ≤ 0.05 (≤2.70 × 10^− 4^) and was used as the threshold to declare whether a significant association signal existed [47].

### Prediction of candidate genes controlling hundred seed weight

Candidate genes, located in the 200-kb flanking genomic region of each peak SNP, were classified and annotated underlying the reference genome. The variation present in exonic regions, splicing sites, 5′UTRs and 3′UTRs, intronic regions, upstream and downstream regions of candidate genes in ten higher HSW lines and ten lower HSW lines was identified from genome re-sequencing data. A gene-based association analysis was conducted using the General Linear Model (GLM) method in TASSEL version 3.0 [46] to identify HSW-related SNPs or haplotypes. Significant SNPs affecting the investigated traits were claimed when the test statistics reached *P* < 0.01.

## Additional files

Additional file 1: Basic genetic parameter statistics for 100-seed weight in the tested soybean population (*n* = 185). (XLS 16 kb)

Additional file 2: Genes in 100 kbp flanking regions of peak SNP associated with 100-seed weight of soybean. (XLS 51 kb)

Additional file 3: Gene-based association study on 100-seed weight of soybean with 281 candidate genes. (XLS 35 kb)

Additional file 4: The information of soybean association panel. (XLSX 16 kb)

Additional file 5: SNP data of 185 soybean accessions for GWAS. (XLSX 1490 kb)

Additional file 6: SNP data of 20 soybean accessions generated from genome resequencing. (XLSX 299 kb)

## Data Availability

All supporting data can be found within the manuscript and its additional files.

## Abbreviations

CATK: The Genome Analysis Toolkit
CMLM: Compressed Mixed Linear Model
CTAB: Hexadecyl trimethyl Ammonium Bromide
GWAS: Genome-wide association studies
HSW: Hundred seed weight
LD: Linkage disequilibrium
LG: Linkage group
MAS: Molecular assistant selection
PCA: The principle component analysis
QTL: Quantitative trait loci
QTN: Quantitative trait nucleotide
RIL: Recombinant inbred line
SLAF-seq: the specific locus amplified fragment sequencing
SW: Seed weight
